# Determining optimal GTV‐to‐PGTV margins for CT‐guided dose‐escalated radiotherapy with daily image guidance in locally advanced rectal cancer

**DOI:** 10.1002/acm2.70429

**Published:** 2025-12-18

**Authors:** Xi Qi, Kai Liu, Yiming Zhang, Weiping Wang, Yangyi Zhang, Yongguang Liang, Zhaoqi Gu, Chen Wang, Wei Zhang, Lecheng Jia, Mengying Yang, Xianhe Zhao, Ke Hu

**Affiliations:** ^1^ Department of Radiation Oncology, Peking Union Medical College Hospital Chinese Academy of Medical Sciences & Peking Union Medical College Beijing China; ^2^ Shanghai United Imaging Healthcare Co., Ltd. Shanghai China

**Keywords:** dose escalation, image‐guided radiotherapy, inter‐fractional motion, PTV margin, rectal cancer

## Abstract

**Background:**

Neoadjuvant chemoradiotherapy for locally advanced rectal cancer yields pathological complete response rates of only 10%–20%. Dose‐escalation strategies may improve outcomes, but optimal GTV‐to‐PGTV margins for CT‐guided radiotherapy with daily IGRT remain undefined.

**Methods:**

Twelve LARC patients undergoing CT‐guided daily IGRT with a simultaneous integrated boost were included. Daily diagnostic‐quality fan‐beam CT (FBCT) scans were acquired for IGRT. GTV and CTV were delineated on planning CT and all FBCTs. Target coverage margins were assessed by isotropically expanding the planning GTV until more than 95% of the voxels of the sequential GTVs were covered. A margin with a coverage probability threshold of 90% was defined as adequate. An independent validation cohort of 30 patients who underwent weekly FBCT‐guided image guidance was further analyzed. Overlap volumes between PGTVs and organs‐at‐risk (OARs; bladder and small bowel) were calculated to assess OAR sparing.

**Results:**

Analysis of 286 FBCT scans showed that a 6 mm isotropic GTV‐to‐PGTV margin achieved>95% coverage in>90% of fractions. Compared with 10 mm expansion, a 6 mm PGTV reduced the overlap volumes with the bladder and small bowel by 68.5% and 68.4%, respectively. A 6 mm isotropic expansion achieved>95% coverage in 91.3% of fractions in the validation cohort.

**Conclusion:**

A 6 mm isotropic GTV to PGTV margin provides adequate target coverage for most middle‐ and lower‐rectal tumors while reducing OAR overlap. This finding could facilitate safer dose escalation while maintaining target coverage. However, larger margins may be necessary for smaller tumors or those located in the high rectum.

## 1 BACKGROUND

Total mesorectal excision(TME) following neoadjuvant chemoradiotherapy is the standard treatment for locally advanced rectal cancer.^1^ Chemoradiotherapy can increase organ preservation rates, improve local control rates, and enhance overall survival. The long‐course radiotherapy regimen is a total dose of 45–50.4 Gy in 25–28 fractions. However, the rates of complete clinical response (cCR) and pathological complete response (pCR) after neoadjuvant chemoradiotherapy are 10%–20%, which is relatively low.^2^ Since the response to radiotherapy is dose‐dependent, increasing the radiation dose to the gross tumor volume (GTV) may improve the pCR rate. This is currently being investigated in a large number of clinical trials, showing that increasing the radiation dose may help to improve the tumor response rate.^3^, ^4^, ^5^

However, increasing the radiation dose is challenging. In modern radiotherapy, rectal cancer patients receive intensity‐modulated radiotherapy (IMRT). IMRT techniques can optimize dose distribution, but are more susceptible to organ motion or deformation. Especially in rectal cancer radiotherapy, intestinal peristalsis and gas in the intestinal lumen can cause significant target motion.^6^ The common approach is to appropriately expand the clinical target volume (CTV) to the planning target volume (PTV), which can reduce the impact of organ motion on the irradiation range. Image‐guided radiation therapy (IGRT) has become an integral part of modern radiotherapy, allowing for visualization and correction of target position before each treatment fraction. Modern IGRT techniques, such as cone‐beam computed tomography (CBCT), enable daily verification and adjustment of patient setup, reducing systematic errors caused by anatomical shifts. Daily IGRT can effectively reduce systematic setup errors and correct for inter‐fractional variations, thereby potentially allowing for smaller PTV margins.^7^ However, there is limited data specifically addressing GTV‐PGTV margins for rectal cancer patients receiving daily IGRT during neoadjuvant chemoradiotherapy.^8^ While the Australasian gastrointestinal trials group (AGTIG) guidelines recommend a three‐dimensional expansion of 0.7‐1.0 cm in the anterior‐posterior direction and 1 cm in the cranial‐caudal direction of the CTV to form the PCTV, these recommendations were not specifically developed for daily IGRT protocols.^9^ Some studies suggest setting different anterior margins based on bladder filling.^10^

For adequate and safe boost dose delivery, a GTV to PGTV margin is needed. Some studies quantify GTV inter‐fraction motion during MR‐guided radiotherapy and give estimates of GTV‐PTV margins.^11^, ^12^ However, there is no consensus on the GTV‐PGTV margin of dose‐escalation strategy using CT‐guided radiotherapy with daily IGRT. Although magnetic resonance‐guided radiotherapy can better display the GTV and has been used in many studies for dose escalation, MR accelerators are not widely used at present. Therefore, it is necessary to develop a margin expansion method based on CT accelerators incorporating daily IGRT. The purpose of this study is to evaluate the inter‐fractional motion of the GTV during CT‐guided radiotherapy with daily IGRT and determine the GTV‐PGTV margin in a simultaneous‐integrated boost (SIB) during five weeks of standard CRT.

## 2 METHODS

### 2.1 Patient eligibility

Twelve patients with rectal cancer, treated with long‐course radiotherapy, were enrolled in this study between October 2023 and March 2024. The patients were staged according to the American Joint Committee on Cancer (AJCC) TNM Staging System for rectal cancer, 8th edition. All patients were confirmed to have adenocarcinoma by pathology. The detailed clinical characteristics are summarized in Table 1. Approval was obtained from the Institutional Review Board of Peking Union Medical College Hospital.

**TABLE 1:** Patient and tumor characteristics.

Patient	Age	Sex	Tumor stage	Nodal stage	Distance from the anal margin(cm)	Tumor longitudinal length(cm)	Tumor circumferential involvement (%)	Pretreatment volume of GTV(cm^3^)	Posttreatment volume of GTV(cm^3^)	Position
1	53	M	T3b	N2b	5.7	6.1	100	334	218	Prone
2	70	F	T3c	N2b	1.8	5.5	75	65	48	Supine
3	68	M	T3b	N2b	8.2	4.3	75	70	31	Supine
4	73	M	T3c	N2b	8.8	3.0	100	45	37	Prone
5	61	F	T3c	N2b	7.8	4.1	50	29	32	Prone
6	69	F	T4b	N0	1.2	4.2	60	47	36	Supine
7	63	F	T4a	N2b	7.5	5.3	100	53	37	Supine
8	57	F	T3c	N2b	7.8	3.7	80	55	54	Supine
9	73	M	T3b	N1b	8.9	4.4	100	58	26	Supine
10	76	M	T4a	N2b	5.8	2.6	50	27	17	Supine
11	62	F	T3b	N1b	6.9	5.4	83	35	23	Supine
12	65	M	T4b	N2b	10.6	10.3	100	323	197	Supine

Abbreviations: F, female and M, male.

In addition, a retrospective validation cohort of 30 patients with locally advanced rectal cancer who underwent weekly FBCT‐guided IGRT during neoadjuvant chemoradiotherapy was analyzed to verify the adequacy of the proposed GTV‐to‐PGTV margins. Each patient received 25 fractions of long‐course radiotherapy with one verification FBCT per week (five scans per patient). Detailed baseline characteristics are provided in Table S1.

### 2.2 Simulation

Three patients underwent simulation in the prone position, and nine patients in the supine position. Simulation used Philips Spectral CT 7500 (Philips Healthcare, Best, The Netherlands) and GE Revolution CT (GE HealthCare, Chicago, IL, USA) with 5 mm slice thickness. Before the simulation, patients were advised to ensure their bladder contained approximately 300–500 mL of urine and to empty their rectum. They were to take 10 mL of meglumine diatrizoate mixed with 200–300 mL of water, which was consumed 1.5 h before the simulation. Those with sufficient renal function received intravenous contrast.

### 2.3 Daily IGRT

Patients were required to prepare the rectum and bladder, similar to the simulation. Prior to each treatment fraction, diagnostic‐quality fan‐beam CT (FBCT) scans were acquired using Philips Spectral CT 7500 (Philips Healthcare, Best, The Netherlands) and GE Revolution CT (GE HealthCare, Chicago, IL, USA). The FBCT scans were performed with the same scanning parameters as the simulation CT. Unlike conventional CBCT, these diagnostic‐quality FBCT images improved soft tissue contrast and minimal image artifacts, enabling precise target visualization. Daily image registration was performed using rigid fusion based on bony anatomy match in three dimensions: left‐right (LR), anterior‐posterior (AP), and superior‐inferior (SI). After registration, couch adjustments were made to correct for setup errors before treatment delivery. The bony anatomy registration was used to eliminate setup‐related displacement. The residual motion assessed in this study, therefore, represented pelvic soft‐tissue deformation after bony alignment correction, which cannot be compensated for by rigid registration.

### 2.4 Target delineation

Due to the poor soft tissue resolution of CT, it is difficult to distinguish tumors from the intestinal wall on CT. In this study, the GTV was defined as the circumferential rectal wall involved by primary tumors. In this study, the tumors of all 12 patients invaded more than 50% of the intestinal circumference, so it was reasonable to take the entire intestinal segment at the primary tumor level as the high‐dose GTV area for dose escalation. This method is also more convenient for the uniform expansion of GTV. The CTV included the primary tumor with the entire mesorectum, presacral space, and regional lymph node regions. For low rectal cancer, the CTV extended inferiorly to include the anal canal and internal anal sphincter. The delineation of CTV and organs at risk was in accordance with the RTOG guidelines.^13^ The bladder and small bowel organ at risk (OAR) were automatically contoured and manually adjusted by radiation oncologists.

### 2.5 Treatment planning

All patients in this study received 6 MV photon Intensity‐Modulated Radiation Therapy. The prescribed dose was delivered using a simultaneous integrated boost (SIB) technique: 45 Gy in 25 fractions to the planning target volume (PTV) and 56 Gy in 25 fractions to the planning gross tumor volume (PGTV).

### 2.6 Margin assessment

The planning GTV expanded isotropically to generate a theoretical PGTV with 1 mm increments from 0 to 10 mm. All CT images (original 5 mm slice thickness) were resampled to isotropic 1 mm voxel grids before analysis, allowing 3D interpolation for volume overlap calculations. The follow‐up GTVs were manually delineated on each FBCT by a radiation oncologist and independently reviewed by a second observer to ensure consistency. Then the planning CT was rigidly aligned to each follow‐up FBCT based on bony anatomy, and the follow‐up GTVs were mapped to the planning CT. GTV coverage was calculated as follows:

$$\begin{array}{cc} & \mathrm{GTV}\;\mathrm{coverage}\left(\%\right)=\\ & \;\frac{\mathrm{PGTV}\;\mathrm{volume}\cap \text{follow-up GTV volume}}{\text{follow-up GTV volume}} \end{array}$$

A follow‐up FBCT GTV was marked as geometrically covered if the coverage is more than 95%. A similar method was also used for CTV margin analysis, where the follow‐up CTV was marked as covered if the coverage is over 98%. This criterion was widely used by previous studies in rectal cancer. An acceptable margin was defined if it geometrically covered over 90% of the total fractions. For each patient, a personal margin was defined if it covered more than 90% of their own fractions. For each FBCT fraction, the overlap volumes between PGTVs (6 and 10 mm isotropic expansions) and OARs were calculated to assess OAR sparing performance.

### 2.7 GTV motion assessment

**FIGURE 1:**
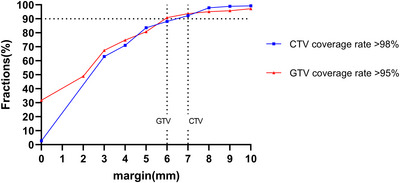
The proportion of fractions reaching the coverage standard for different PTV margins. For GTV, a margin greater than 6 mm can meet the 95% coverage standard in more than 90% of the total fractions. For CTV, a margin greater than 7 mm can meet the 98% coverage standard in more than 90% of the total fractions.

To further evaluate GTV motion, all fractional FBCTs were rigidly aligned to the planning CT based on bony anatomy. All delineations were projected on the planning CT, as shown in Figure 1.

For all the aligned delineation, the centroid of GTVs was determined with the software 3D slicer.^14^ Next, the left/right (LR), anterior/posterior (AP), and cranial/caudal (CC) distances between the centroid of planning GTV and the centroids of all the FBCTs scans were determined. Mean and standard deviation of these distances were calculated for each patient.

### 2.8 Statistics

The mean, median, range, and standard deviation of the displacement of the GTV volume in each patient were calculated. All statistical analysis and processing were conducted using SPSS software, version 23.0 (IBM Corp, Armonk, NY, USA). Two‐sided *p*‐values < 0.05 were considered statistically significant. In cases where the data adhered to the normal distribution, the paired t‐test was used for statistical analysis; alternatively, the Wilcoxon rank sum test was utilized when normality assumptions were not met.

## 3 RESULTS

### 3.1 Clinical characteristics of patients

Twelve patients were included in this study, who were treated with long‐course radiotherapy (25*2 Gy, LCRT) between October 2023 and March 2024. Patient and tumor characteristics are summarized in Table 1. One patient had a distal rectum tumor (tumor distance from anal margin 10∼15 cm), 9 patients had a middle rectum tumor (tumor distance from anal margin 5∼10 cm), and 2 patients had a lower rectum tumor (tumor distance from anal margin 0∼6 cm). The median planning GTV volume was 54 cm^3^ with an IQR of 37.5–68.75 cm^3^.

A total of 300 FBCT scans were obtained from 12 patients. Some scans were excluded due to artifacts or technical issues, such as low imaging quality, large respiratory amplitude. Consequently, a total of 286 FBCT scans were analyzed, with an average number of scans per patient of 23.9.

### 3.2 Target coverage and planning margin

**FIGURE 2:**
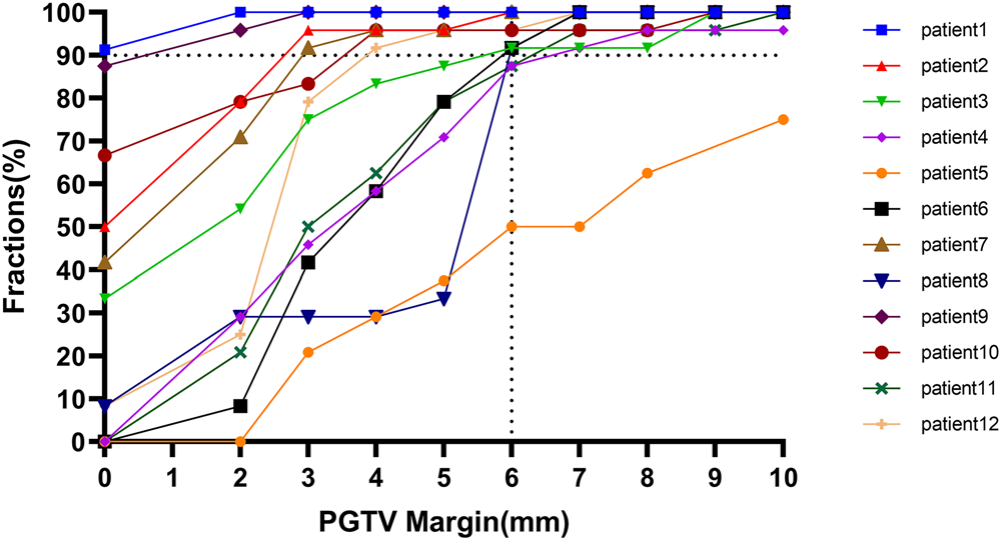
Schematic overview of the required PGTV margins per patient. Margins were defined as adequate for a patient when 90% of this patient's follow‐up GTVs were covered by over 95% by PGTV.

As shown in Figure 1, for GTV, a margin greater than 6 mm can cover more than 90% of the fractions in all cases (261/286). For CTV, a margin greater than 7 mm can cover more than 90% of the fractions (265/286). As shown in Figure 2, among the 12 patients, 9 patients can cover more than 90% of the fractions when using a 6 mm GTV‐PGTV margin. A 7 mm GTV‐PGTV margin can cover more than 90% of the fractions in 91% of patients (11/12). When GTV is expanded by 1 cm, all fractions of the remaining 10 patients can be covered except for patient 4 and patient 5 (Figure S1). For patient 4, only one fraction was not covered by a 1 cm expansion because of gas filling in the rectal lumen. However, for patient 5, even when GTV is expanded by 1 cm, 25% of the fractions (6/24) still fail to meet the coverage standard. This is due to the small GTV size (29 cm^3^) and the huge motion of the tumor in this patient.

**FIGURE 3:**
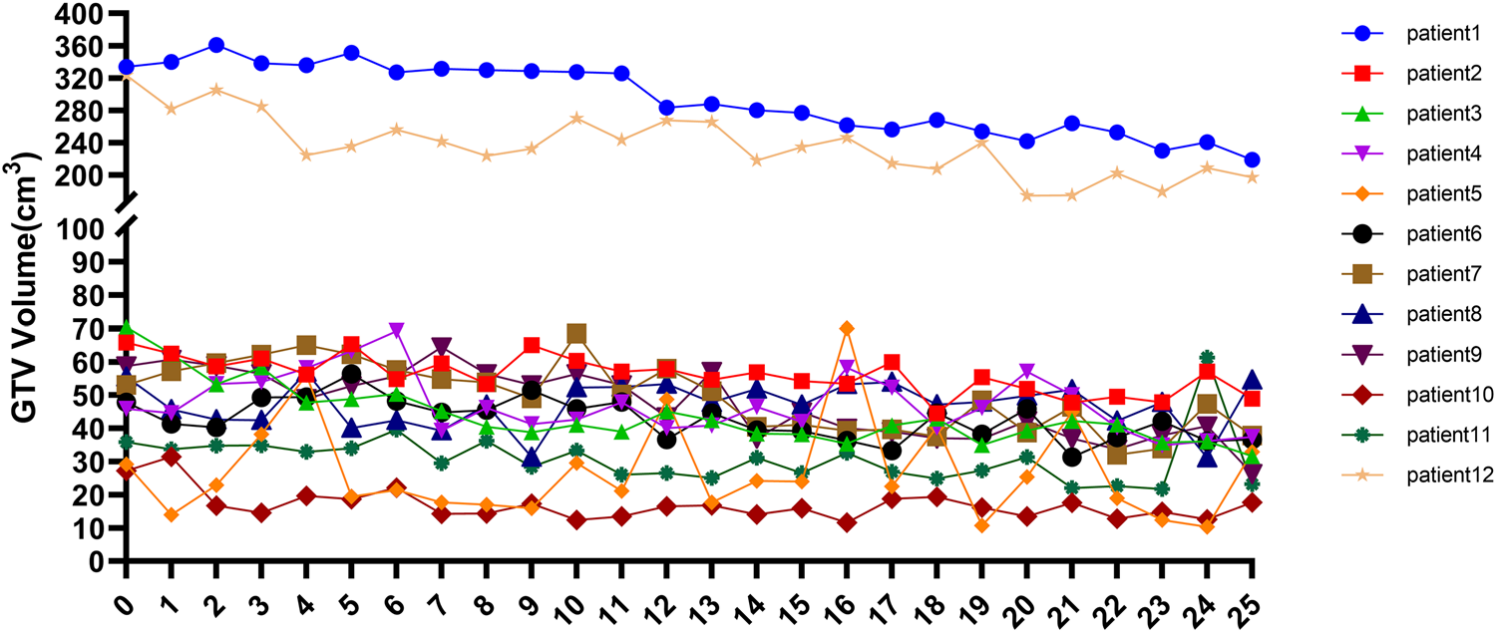
The GTV volumes throughout the treatment for each individual patient.

We then sought to estimate the change in GTV volumes during the therapy. As shown in Figure 3, the pretreatment GTV volumes of patients 1 and 12 were larger than those of the other patients. The average pretreatment GTV volume of these two patients was 328.5 cm^3^, while the average for other patients was 48.4 cm^3^. Meanwhile, the GTV volume of these two patients decreased during the treatment process. The average percentage and volume decrease were 36% and 121 cm^3^ compared with the pretreatment GTV. This led to better coverage of GTV by the planning GTV. For patient 1, the planning GTV could cover more than 90% of the fractions (21/23) even without any expansion, and for patient 12, an expansion of 4 mm could cover more than 90% of the fractions (22/24). The other 10 patients, although the relative GTV volume reduction was statistically significant (*p* < 0.01), the absolute decrease was modest (14.3 cm^3^). We also analyzed the fractions that failed to meet the 6 mm coverage criterion. The median fraction number at which coverage failure occurred was 14 (IQR: 3–17), indicating that these events were evenly distributed throughout the treatment course.

### 3.3 Validation of GTV margin

To validate these findings, an additional cohort of 30 patients treated with weekly FBCT‐guided IGRT was analyzed (150 fractions in total). A 6 mm isotropic GTV expansion achieved > 95% volume coverage in 137 fractions (91.3%), while a 7 mm expansion achieved > 95% coverage in 144 fractions (96.0%). At the patient level, 5 of 30 patients had one fraction not meeting the standard with 6 mm expansion, and 4 patients had two fractions not meeting the standard. These four patients had significantly higher tumor locations (mean tumor distance from the anal verge 8.63 vs. 6.70 cm, *p* = 0.006). With 7 mm expansion, only 2 patients had one, and 2 had two substandard fractions. These results further confirm that 6–7 mm isotropic margins provide sufficient target coverage under daily image guidance.

### 3.4 Evaluation of GTV motion

**FIGURE 4:**
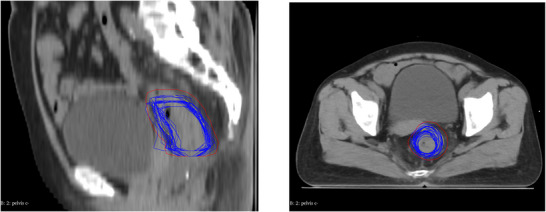
GTV displacement of patient 8. Both the sagittal (left) and axial (right) views are taken at the level of the maximum tumor volume. The planning GTV (yellow) is delineated on the planning CT, and PGTV (red) is generated with a 6 mm expansion. All the GTVs (blue) contour from the FBCT were mapped to the planning CT after rigid registration with respect to bony anatomy.

To further validate the 6 mm margin determined in our coverage analysis, we evaluated the specific GTV displacement patterns. Figure 4 shows the GTV movement between fractions of the patient by transforming the GTV of 25 fractions onto the planning CT. The GTV of the planning CT is delineated in yellow, and the GTV of each fraction is delineated in blue. As evident from Figure 4, the 6 mm expansion margin provides adequate coverage in nearly all fractions for patient 8, confirming our previous coverage analysis results. Quantitative assessment of centroid movement showed that patient 8 had an average anterior‐posterior, lateral, and superior‐inferior shifts with standard deviations of 1.25 ± 2.32 mm, 2.04 ± 1.44 mm, and 1.82 ± 2.28 mm, respectively, representing typical motion patterns in our cohort. In contrast, patient 5, who required larger margins in our previous analysis, had an average anterior‐posterior, lateral, and superior‐inferior shifts with standard deviations of 5.84 ± 3.38 mm, 0.58 ± 5.03 mm, 4.41 ± 4.36 mm, respectively, which showed significantly greater motion compared to other patients (Figure S2). These quantitative measurements explain why the 6 mm margin failed to provide adequate coverage for patient 5.

### 3.5 Organ‐at‐risk analysis

Among the 286 fractions, PGTV_10 mm overlapped with the bladder in 175 fractions and with the small bowel in 188 fractions, whereas PGTV_6 mm overlapped with the bladder in 130 fractions and with the small bowel in 158 fractions. For fractions showing overlap, the median overlapping volume between PGTV_10 mm and the bladder was 7.28 cm^3^ (IQR: 1.88–24.22 cm^3^), while that between PGTV_6 mm and the bladder was 2.37 cm^3^ (IQR: 0.10–14.00 cm^3^), representing an average 68.5% reduction (95% CI: 64.3%–72.7%). Similarly, for the small bowel, the median overlapping volume for PGTV_10 mm was 7.35 cm^3^ (IQR: 2.47–12.80 cm^3^), compared to 2.57 cm^3^ (IQR: 0.20–5.42 cm^3^) for PGTV_6 mm, corresponding to an average 68.4% reduction (95% CI: 65.4%–71.5%). These results indicate that 6 mm isotropic expansions substantially reduce overlap with adjacent OARs while maintaining adequate target coverage.

## 4 DISCUSSION

This study is the first to propose an appropriate PTV expansion margin for rectal cancer LCRT with simultaneous dose escalation using CT‐guided radiotherapy with daily IGRT. We evaluated the inter‐fraction GTV and CTV motion over time in a group of rectal cancer patients and simulated the coverage of the target area under different expansion margins to assess margins needed for a CT‐guided dose‐escalation strategy. We found that the isotropic margin needed to cover 90% of inter‐fraction GTV motion was 6 mm. When applying a 7 mm margin, all patients except one with a small‐volume rectal tumor achieved adequate coverage (> 95% volume) in more than 90% of their treatment fractions. The isotropic margin needed to cover 90% of inter‐fraction CTV motion was 7 mm. From an anatomical standpoint, the 6–7 mm isotropic margin significantly reduced the overlap volumes with the bladder and small bowel by approximately 68%, compared with a 10 mm expansion. This finding demonstrates that tighter margins can meaningfully reduce OAR exposure without compromising target coverage, supporting safer implementation of dose‐escalated protocols.

Although limited in number, prior studies have attempted to systematically evaluate rectal cancer GTV motion and deformation during RT. Yamashita et al. analyzed rectal motion during preoperative chemoradiotherapy using weekly CBCT scans and found that when applying margins of 0, 3, 5, 7, 10, and 15 mm to the rectum, the mean percentages of rectum exceeding the target volume were 20.7%, 7.2%, 3.9%, 2.1%, 0.7%, and 0.1%, respectively.^15^ Their results were comparable to our findings, despite methodological differences. Brierley et al. investigated week‐to‐week inter‐fraction motion in rectal cancer based on CT,^16^ they concluded that the GTV expansion margins in different directions were 8 mm right, 7 mm left, 14 mm anterior, and 7 mm posterior, which were significantly higher than our results for the anterior margin and slightly higher than our results for other directions. This might be explained by the lower number of samples per patient (3 per patient in their study, 25 per patient in our study). Meanwhile, they calculated a system and random error based on the changes of GTV boundaries to generate the expansion margins in different directions. In our study, PGTV was generated by uniformly expanding GTV, and the spatial coverage of PGTV over GTV was calculated. A margin was considered sufficient if it covered more than 95% of the volume, with more emphasis on the overall coverage rather than the movement in a single direction, thus resulting in a uniform expansion margin. In recent years, most studies on dose escalation for rectal cancer have adopted the method of uniformly expanding GTV.^17^, ^18^ Additionally, a lower coverage standard can be used for GTV because GTV will be irradiated as an enhancement to CTV, leading to a less steep dose gradient.

Hidde et al.^18^ studied the inter‐fractional motion of GTV in MRI‐guided dose‐escalated radiotherapy for rectal cancer and found that a 17 mm margin could cover GTV in 90% of the fractions, which is much larger than the 6 mm margin in our study. Their study delineated GTV as the primary tumor shown on MRI, rather than the entire intestinal segment where the tumor was located, as in this study. Moreover, the patients in their study were at an earlier stage (47% were at the T2 stage). Therefore, the median GTV volume in their study was only 12.1 cm^3^, significantly smaller than the 54 cm^3^ in this study. Previous studies have found that larger tumors have less inter‐fractional motion. Additionally, compared to 91% of low‐middle rectal cancer patients in this study, the proportion of low‐middle rectal cancer patients in their study was relatively low (40%), and proximal might be more vulnerable to being affected by bladder filling. Van de Ende et al. studied the inter‐ and intrafraction displacement of the GTV based on fiducial markers on cone beam CT images and found that tumors located in the mid and upper rectum, inter‐fraction errors were up to 9.4 mm (systematic) and 5.6 mm (random) compared with 4.9 and 2.9 mm for tumors in the lower rectum.^19^ Chong et al.^20^ analyzed the motion by dividing the rectum into upper, middle, and lower parts and found that rectal motion was smallest in the lower part. Our results concurred with these observations. In our validation cohort, we found that patients with inadequate 6 mm isotropic margins had significantly higher tumor locations, suggesting that high‐rectal tumors are subject to greater motion, likely due to reduced mesorectal constraint.

Limitations of our study include its relatively small sample size. To mitigate this limitation, an additional validation analysis including 30 patients with weekly FBCT‐guided IGRT was performed, confirming the adequacy of 6–7 mm isotropic margins. Nevertheless, larger multi‐institutional studies are warranted to establish population‐based margin recommendations. Additionally, while our findings are robust for centers implementing daily IGRT protocols, they may not be generalizable to institutions using different image guidance frequencies or technologies. Furthermore, our study focused mainly on middle‐lower rectal tumors with large tumor volume; the proposed margins may not be suitable for tumors in other locations.

Currently, there is no consensus on the GTV‐PGTV margin used in dose escalation studies based on CT‐guided radiotherapy.^21^, ^22^, ^23^ Our result shows that reducing the GTV‐PGTV margin to 6 mm is feasible when dose escalation is based on CT‐guided radiotherapy with daily IGRT. A smaller margin may allow for higher boost doses and lower doses to OARs. Most of the patients included in this study were at a later stage and had larger tumor volumes of low rectal cancer. These patients may benefit from dose‐escalation radiotherapy. For patients with smaller tumors and those located in the upper rectum, a 6 mm margin may be insufficient, and MRI‐guided radiotherapy or adaptive radiotherapy may be more suitable for these patients. Furthermore, centers unable to implement daily IGRT should consider using larger margins or alternative strategies for dose escalation.

## AUTHOR CONTRIBUTIONS

*Conception and design*: Ke Hu, Xi Qi, and Kai Liu. *Collection and assembly of data*: Xi Qi, Weiping Wang, Yiming Zhang, Yangyi Zhang, Yongguang Liang, Zhaoqi Gu, Chen Wang, and Xianhe Zhao. *Data analysis and interpretation*: Xi Qi, Wei Zhang, Lecheng Jia, and Mengying Yang. All the authors contributed to the writing and final approval of the manuscript.

## CONFLICT OF INTEREST STATEMENT

The authors declare that they have no known competing financial interests or personal relationships that could have appeared to influence the work reported in this paper.

## ETHICS APPROVAL AND CONSENT TO PARTICIPATE

The Institutional Review Board (IRB) of Peking Union Medical College Hospital reviewed the protocol. This is a retrospective study. The protocol is rational and scientific. The study is in accordance with ethical principles. The IRB thus approves the protocol.

## Supporting information



Supporting information

Supporting information

## Data Availability

The datasets used and analyzed during the current study are available from the corresponding author on reasonable request.
