# Supplementary material for: Determining optimal GTV‐to‐PGTV margins for CT‐guided dose‐escalated radiotherapy with daily image guidance in locally advanced rectal cancer
Source: J Appl Clin Med Phys. 2025 Dec 18;27(1):e70429. doi: 10.1002/acm2.70429 (PMC12715369; doi:10.1002/acm2.70429)
Supplement: Supplementary file 1 — Supporting information [file ACM2-27-e70429-s001.docx]

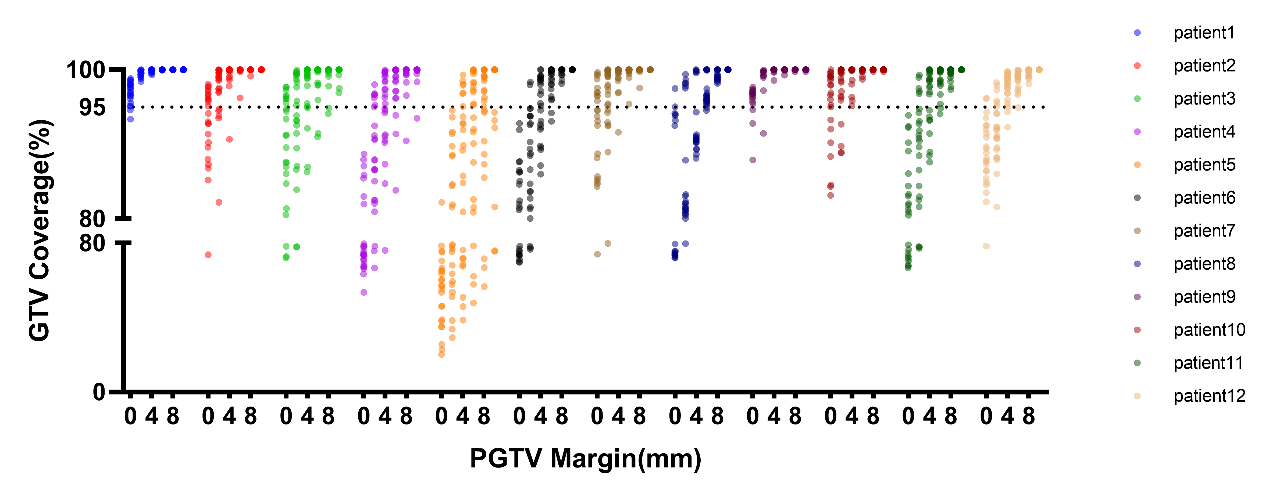


**Supplementary Fig 1. GTV coverage for each fraction with 0,2,4,6,8,10 mm margin in different patients.** A follow-up fraction’s GTV was marked as geometrically covered if the coverage is more than 95%.


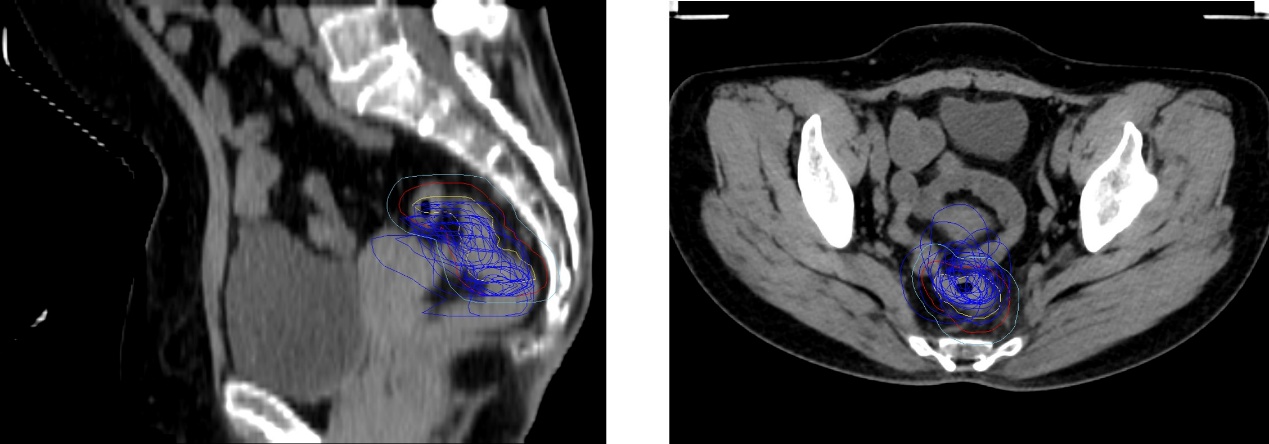


**Supplementary Fig 2. GTV displacement of patient 5.** Both the sagittal (left) and axial (right) views are taken at the level of the maximum tumor volume. The reference GTV (yellow) is delineated on planning CT, 6 mm margin PGTV (red) and 10 mm margin PGTV (light blue) are shown. All the GTVs (dark blue) contour from the FBCT were mapped to the planning CT after rigid registration with respect to bony anatomy.
