# Supplementary material for: Determining optimal GTV‐to‐PGTV margins for CT‐guided dose‐escalated radiotherapy with daily image guidance in locally advanced rectal cancer
Source: J Appl Clin Med Phys. 2025 Dec 18;27(1):e70429. doi: 10.1002/acm2.70429 (PMC12715369; doi:10.1002/acm2.70429)
Supplement: Supplementary file 2 — Supporting information [file ACM2-27-e70429-s002.docx]

**Supplementary Table 1. Characteristics of validation patients (n=30)**

| Characteristics | No. of patients (n,%) |
| --- | --- |
| **Age (median, IQR), y**  **Sex**  Male  Female  **T stage**  T3  T4  **N stage**  N0  N1  N2  **Distance from anal margin (median, IQR), cm**  0~5  5~10  **GTV volume (median, IQR), cm^3^** | 62.5 (47-68)  25 (83.3)  5 (16.7)  20 (66.7)  10 (33.3)  3 (10)  10 (33.3)  17 (56.7)  7.0 (5.9-8.0)  3 (10)  27 (90)  56 (36-73) |
